# Quinary wurtzite Zn-Ga-Ge-N-O solid solutions and their photocatalytic properties under visible light irradiation

**DOI:** 10.1038/srep19060

**Published:** 2016-01-12

**Authors:** Yinghao Xie, Fangfang Wu, Xiaoqin Sun, Hongmei Chen, Meilin Lv, Shuang Ni, Gang Liu, Xiaoxiang Xu

**Affiliations:** 1 Shanghai Key Lab of Chemical Assessment and Sustainability, Department of Chemistry, Tongji University, 1239 Siping Road, Shanghai, 200092, China; 2 Science and Technology on Plasma Physics Laboratory, Laser Fusion Research Center, China Academy of Engineering Physics, Mianyang 621900, China; 3 Shenyang National laboratory for Materials Science, Institute of Metal Research, Chinese Academy of Science, 72 Wenhua Road, Shenyang 110016, China

## Abstract

Wurtzite solid solutions between GaN and ZnO highlight an intriguing paradigm for water splitting into hydrogen and oxygen using solar energy. However, large composition discrepancy often occurs inside the compound owing to the volatile nature of Zn, thereby prescribing rigorous terms on synthetic conditions. Here we demonstrate the merits of constituting quinary Zn-Ga-Ge-N-O solid solutions by introducing Ge into the wurtzite framework. The presence of Ge not only mitigates the vaporization of Zn but also strongly promotes particle crystallization. Synthetic details for these quinary compounds were systematically explored and their photocatalytic properties were thoroughly investigated. Proper starting molar ratios of Zn/Ga/Ge are of primary importance for single phase formation, high particle crystallinity and good photocatalytic performance. Efficient photocatalytic hydrogen and oxygen production from water were achieved for these quinary solid solutions which is strongly correlated with Ge content in the structure. Apparent quantum efficiency for optimized sample approaches 1.01% for hydrogen production and 1.14% for oxygen production. Theoretical calculation reveals the critical role of Zn for the band gap reduction in these solid solutions and their superior photocatalytic acitivity can be understood by the preservation of Zn in the structure as well as a good crystallinity after introducing Ge.

Photocatalytic hydrogen production from water in the presence of semiconductor is a simple and tempting approach for solar energy storage into chemical fuels. Ideally, such a process includes a simple uphill reaction that water molecules are split into hydrogen and oxygen at the surface of a semiconductor, driven solely by incoming photons. Materials suitable for this reaction have to be light sensitive and catalytically active simultaneously, which by far is subject to only a few compounds or systems^1^,^2^,^3^,^4^,^5^,^6^,^7^,^8^,^9^,^10^. In particular, complete water splitting using visible light photons (λ ≥ 400 nm) is only reported in some metal oxides/oxynitrides containing elements with *d*^10^ electronic configurations^11^,^12^,^13^ and an organic semiconductor g-C_3_N_4_ with carbon nanodots^14^. Of note is inorganic solid solutions (Ga_1-x_Zn_x_)(N_1-x_O_x_) with wurtzite structure that exhibit a quantum efficiency (QE) for overall water splitting as high as 2.5% at 420–440 nm^1^. Apart from its high QE, their photocatalytic performance were found to be highly structural and compositional dependent that are strongly controlled by a number of factors such as starting materials, Zn/Ga ratio, nitridation temperature, nitridation time, ammonia flow rate and cocatalyst etc^13^,^15^,^16^,^17^,^18^,^19^,^20^,^21^,^22^,^23^,^24^,^25^,^26^,^27^,^28^,^29^,^30^. Care has to be taken during the preparation and optimization of these solid solutions but compositional discrepancies within individual particles^13^ as well as split anion sites in their structure^30^ are still encountered. This could be largely attributed to the volatile nature of Zn species in the presence of ammonia at high temperatures that most Zn is lost from the system due to reduction and evaporation^31^. For this reason, a large excess amount of Zn has to be used during the synthesis in order to compensate Zn loss. For instance, a Zn/Ga ratio around 0.1 in the final product often require a starting Zn/Ga molar ratio higher than 1^13^,^21^,^28^. More importantly, previous investigations revealed that optical properties, crystallinity and photocatalytic performance of (Ga_1-x_Zn_x_)(N_1-x_O_x_) solid solutions are substantially governed by Zn content in the structure^13^,^17^,^21^,^30^,^32^. Higher amounts Zn in the solid solution normally guarantees more light absorption and a better crystallinity, thereby a higher photocatalytic activity^13^,^28^,^32^. Thus, management of Zn quantity in the solid solution is of critical importance in developing active photocatalysts. Current optimal synthetic procedures normally involve a high Zn/Ga starting ratio (>1) and a prolonged nitridation time (≥15 h) with a high ammonia flow rate (≥250 ml/min), which is costly and time-consuming, probably a compromise between Zn evaporation and crystal growth^13^,^21^,^30^,^32^. Here, we systematically investigated a quinary metal oxynitrides solid solutions Zn-Ga-Ge-N-O by introducing Ge into (Ga_1-x_Zn_x_)(N_1-x_O_x_) solid solutions. The high positive charge of Ge cation (+4) might be helpful for the stabilization of Zn (+2) in the wurtzite structure. The effects of adding Ge in the wurtzite structure were carefully studied in terms of phase formation, microstructure, optical and photocatalytic properties. Combined with theoretical calculation and various surface analyzing techniques, possible mechanism was explored. Our results suggest that the presence of Ge in the solid solution significantly prohibit Zn volatilization and promote crystallization of the compound, resulting in a shorter nitridation time. The prepared Zn-Ga-Ge-N-O compounds demonstrate promising photocatalyic activity under the optimized synthetic conditions.

## Results and Discussions

### Phase purity and ammonolysis conditions

**Figure 1:**
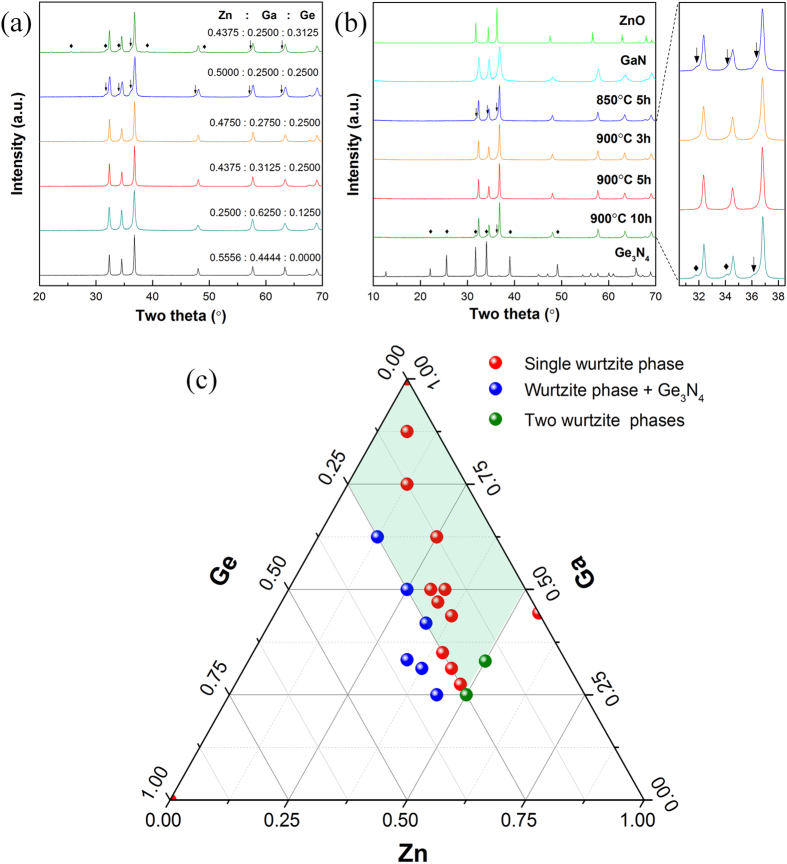
X-ray powder diffraction patterns of as-prepared samples: (**a**) samples with different starting Zn/Ga/Ge molar ratios under the same nitridation conditions (900 °C for 5 hours); (**b**) samples with fixed starting Zn/Ga/Ge molar ratio (0.4375: 0.3125: 0.2500) nitridized at different temperatures and time, patterns of GaN and Ge_3_N_4_ were shown for comparisons. Impurities were labeled as ♦ for Ge_3_N_4_ and ↓ for secondary wurtzite phase; (**c**) Relationship between starting Zn/Ga/Ge molar ratios and phase compositions of the synthetic powders at fixed nitridation conditions (900 °C for 5 hours with ammonia flow rate ~250 ml/min).

As Zn is highly volatile in current synthetic conditions (ammonia and high temperatures), we have carried out a systematic investigation on the preparation conditions of this quinary solid solutions. Starting molar ratio of Zn, Ga and Ge as well as calcining temperatures and time are examined. Composition of synthetic powders was analyzed using XRD techniques. The results are shown in Fig. 1 and detailed information are summarized in Table 1S. From XRD analysis, compositions of the final products are strongly dependent on starting Zn/Ga/Ge molar ratio, ammonolysis temperature and time. Single phase with up to 25% Ge in the starting powders can be realized by carefully controlling the initial Zn dosage. All peaks can be indexed with a hexagonal symmetry; therefore, solid solutions between Zn, Ga and Ge were successfully prepared. However, a low temperature (for example 850 °C) and a long time for ammonolysis (10 hours) normally lead to the presence of impurities (Fig. 1a,b). *β*-Ge_3_N_4_ appears if starting Ge dosage exceeding 25% or a long ammonolysis time. Secondary phase with similar hexagonal symmetry comes out if too much Zn is introduced in the starting powders or a low ammonolysis temperature. This secondary phase is probably also a solid solution rather than unreacted ZnO as it is resistant to acid washing. We then fix the optimal temperature and time for ammonolysis (900 °C and 5 hours) in order to clarify the effect of starting Zn/Ga/Ge molar ratio. The results are summarized in Fig. 1c. Formation of single phase (red dot) is generally limited to the conditions that Ge dosage is less than 25% and Zn dosage is less than 50% in the starting powders (shaded parallelogram area). However, higher Zn dosage (>50%) is often needed for a single phase formation without Ge, as previously studied in the literatures^13^,^21^.

On the other hand, Rietveld refinement of XRD patterns revealed that the so-formed single phase is indeed a solid solution that has a wurtzite structure (space group *P* 6_3_*mc* (No.186)). Good refinement was achieved by adding constrains that Zn/Ga/Ge cations occupy the same position as well as N/O anions. Supplementary Fig. S1 shows the typical Rietveld refinement of XRD patterns from product (Entry 12 in Supplementary Table 1S). The refinements converged with good *R*-factors and χ^2^ (*R*_*p*_ = 6.53%, *R*_*wp*_ = 8.38 and χ^2^ = 2.808). Neither refining the thermal parameters of O/N ions nor releasing the constraint on the position of Zn/Ga/Ge and N/O led to any significant improvement in the *R* factors. Therefore, accommodation of cations Zn/Ga/Ge and anions N/O are most likely random in the structure. In addition, all solid solutions have much smaller unit cell parameters than ZnO, presumably due to the mixing of smaller Ga and Ge cations with large Zn cations at the same position (ionic radii for Zn^2+^, Ga^3+^ and Ge^4+^ are 0.6 Å, 0.47 Å and 0.4 Å at coordination number of 4)^33^ according to Vegard’s law. The refined unit cell parameters are listed in Supplementary Table S2. Formation of a single phase was also supported from TEM analysis (Supplementary Figs S2 and S3).

### Microstructures

**Figure 2:**
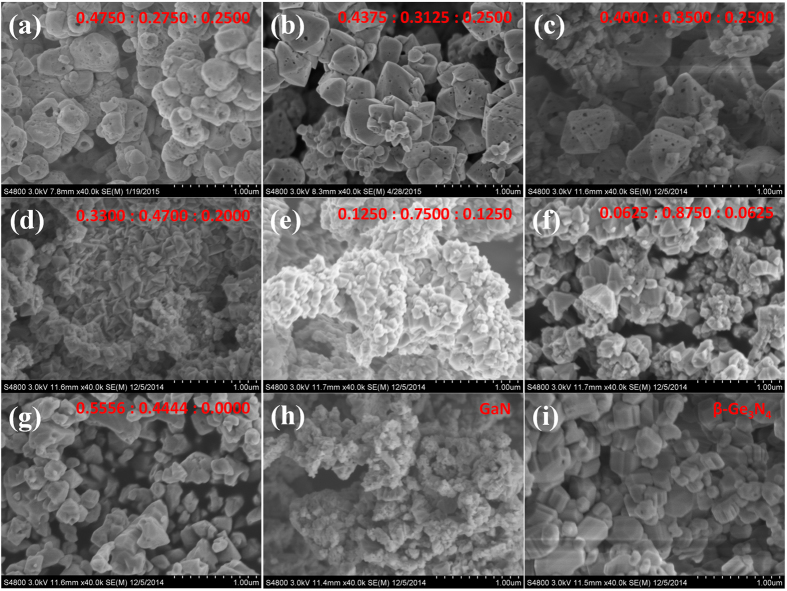
Field emission scanning electron microscopy images of samples prepared from different starting Zn/Ga/Ge molar ratios: (**a**) 0.4750: 0.2750: 0.2500, (**b**) 0.4375: 0.3125: 0.2500, (**c**) 0.4000: 0.3500: 0.2500, (**d**) 0.3300: 0.4700: 0.2000, (**e**) 0.1250: 0.7500: 0.1250, (**f**) 0.0625: 0.8750: 0.0625, (**g**) 0.5556: 0.4444: 0.0000, (**h**) 0.0000: 1.0000: 0.0000, i.e. GaN and (i) 0.0000: 0.0000: 1.0000, i.e. Ge_3_N_4_. Ammonolysis conditions were all kept the same at 900 °C for 5 hours with ammonia flow rate ~250 ml/min.

The crystallinity of samples were found to be the critical factor for high photocatalytic activity during the investigation of (Ga_1-x_Zn_x_)(N_1-x_O_x_) solid solution^13^,^28^. Using different type of raw materials like α-Ga_2_O_3_, β-Ga_2_O_3_ and γ-Ga_2_O_3_ or even pretreatment of ZnO would induce considerable variations to the photocatalytic performance of the final products. Microstructure analysis reveals a strong correlation between high crystallinity and high catalytic activity. However, particles of (Ga_1-x_Zn_x_)(N_1-x_O_x_) solid solution under SEM conditions all appear to be irregular, even under optimized synthetic procedures^9^. This was also confirmed in our study that sample prepared by nitridizing Ga_2_O_3_ and ZnO mixtures contains featureless particles, ranging from hundred nanometers to micron in diameter (Fig. 2g). A striking difference in the particle morphology was noticed after introducing Ge to the starting materials. Sharp crystal edges and smooth crystal planes are clearly identified, even with addition of trivial amounts of Ge (Fig. 2f). Germanium, therefore, strongly promote the crystallization of solid solutions. Substantial growth of particles is more evident in samples with high starting Ge molar ratios and their microstructures are dominated by double-pyramid shaped particles (Fig. 2b,c). Best recipe for preparing these double-pyramid shaped particles are Zn/Ga/Ge = 0.4375/0.3125/0.2500 in the starting mixtures. Nevertheless, further increasing the usage of Zn seems to have negative effect on the particle crystallinity as such particle morphology is lost at higher Zn molar ratio (Fig. 2a). It is interesting to see some small pinholes at the surface of these double-pyramid shaped particles, probably due to the evaporation of Zn from the crystals. The reasons for Ge to be an effective promotor for crystallization are not clear, but can be referred to the facile growth of Ge_3_N_4_ particles at the same synthetic conditions. Figure 2i shows the SEM images of GeO_2_ ammonolysed at 900 °C for 5 hours, large cylinder shaped particles are clearly visible, indicative of good particle growth. Conversely, Ga_2_O_3_ ammonolysed at the same conditions displays agglomerates of nanoparticles. These observations are consistent with XRD patterns in Fig. 1b where Ge_3_N_4_ has narrow and shape peaks whereas GaN contains only broad peaks.

**Figure 3:**
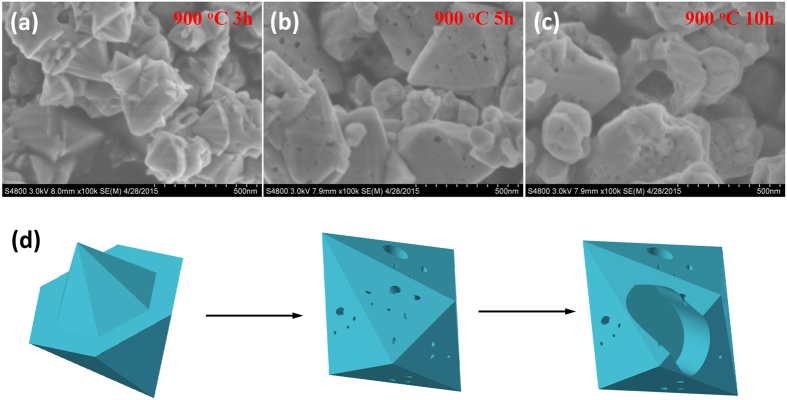
Field emission scanning electron microscopy images of samples prepared under different ammonolysis time. (**a**) 3 h, (**b**) 5 h, (**c**) 10 h and (**d**) schematic show of particle growth. Starting Zn/Ga/Ge molar ratios were fixed (0.4375: 0.3125: 0.2500) and ammonolysis temperature is 900 °C with constant ammonia flow rate ~250 ml/min

To better understand the history of particle growth, we then take a close examination of samples underwent different ammonolysis time. Figure 3 shows SEM images of samples (Zn/Ga/Ge = 0.4375/0.3125/0.2500) ammonlysed for different time at 900 °C. It can be seen from the images that crystal growth has been occurring substantially even for a short ammonlysed time (3 hours). Sharp crystal edges and smooth crystal planes are clearly visible and seem to have features of crystal twinning phenomenon (Fig. 3a,d)^34^. Complete crystal growth was likely achieved after additional 2 hours according to their particle appearance that is symmetric and pyramid shaped. Nonetheless, pinholes due to Zn evaporation already become visible at this stage, confirming the volatile nature of Zn at ammonlysis conditions (Fig. 3b). It has been suggested that ZnO is reduced to metallic Zn after exposing to ammonia atmosphere, which then melts into liquid phase above 420 °C^21^. The high temperature used here (900 °C) is very close to the boiling point of Zn (b.p. = 907 °C), therefore a severe evaporation of Zn is expected^35^. This is more clearly seen after longer ammonolysis time (10 hours) that leads to the formation of large holes and hollow particles (Fig. 3c). The loss of Zn is also confirmed from XRD analysis that Ge segregate out from the solid solution according to the *β*-Ge_3_N_4_ peaks (Fig. 1b). Therefore, ammonolysis time is one of the critical factors for particle crystallinity, with short time for incomplete particle growth and long time for particle degradation. The best ammonolysis time presumably lies around 5 hours.

### UV-vis spectra

**Figure 4:**
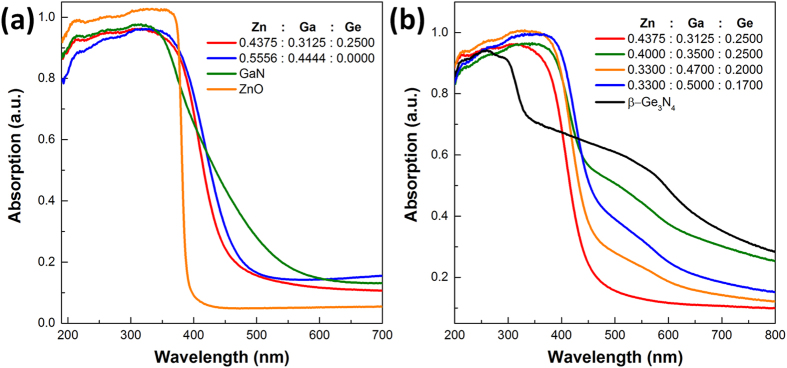
UV-visible light absorption spectra (converted from diffuse reflectance spectra) of (**a**) samples with or without Ge in the raw materials, GaN and ZnO are included for comparisons; (**b**) samples with different starting Ge molar ratio, *β*-Ge_3_N_4_ is shown for comparisons.

UV-visible diffuse reflectance spectra of as-prepared samples are shown in Fig. 4, along with ZnO and GaN or *β*-Ge_3_N_4_ for comparison. Solid solutions, either with or without Ge demonstrate apparent red shift of their absorption edges compared with ZnO, indicating visible light absorption. The absorption curve of (Ga_1-x_Zn_x_)(N_1-x_O_x_) solid solution is very similar to the results reported in the literatures^13^,^21^. Typical Zn-Ga-Ge-N-O solid solution has a band gap about 2.8 eV, close to the value 2.7 eV of (Ga_1-x_Zn_x_)(N_1-x_O_x_) solid solution (Fig. 4a). Incorporating different amounts of Ge into the wurtzite structure would slightly alter the absorption edges (Fig. 4b). It is interesting to see GaN prepared under ammonolysis conditions exhibits a long absorption tail into visible light region. Considering the intrinsic band gap of GaN ~3.4 eV, this tail is likely due to defect levels induced during synthesis^36^. Similar observation is also noticed in case of *β*-Ge_3_N_4_, in which absorption extends well into infrared region (Fig. 4b). This absorption tail has been attributed to the reduced Ge species (Ge^0^, Ge^2+^) and is not involved in the photocatalytic processes^37^,^38^. Such absorption tail is also discernable in the solid solutions containing Ge. The only exception is the sample with highest crystallinity (starting Zn/Ga/Ge = 0.4375/0.3125/0.2500) (see Fig. 4b red line). Defects generally act as charge recombination centers in the structure therefore deteriorate photocatalytic performance. The lack of Ge type defects in the solid solution is strongly desired for the achievement of high activity.

### X-ray photoelectron spectra and atomic composition

**Figure 5:**
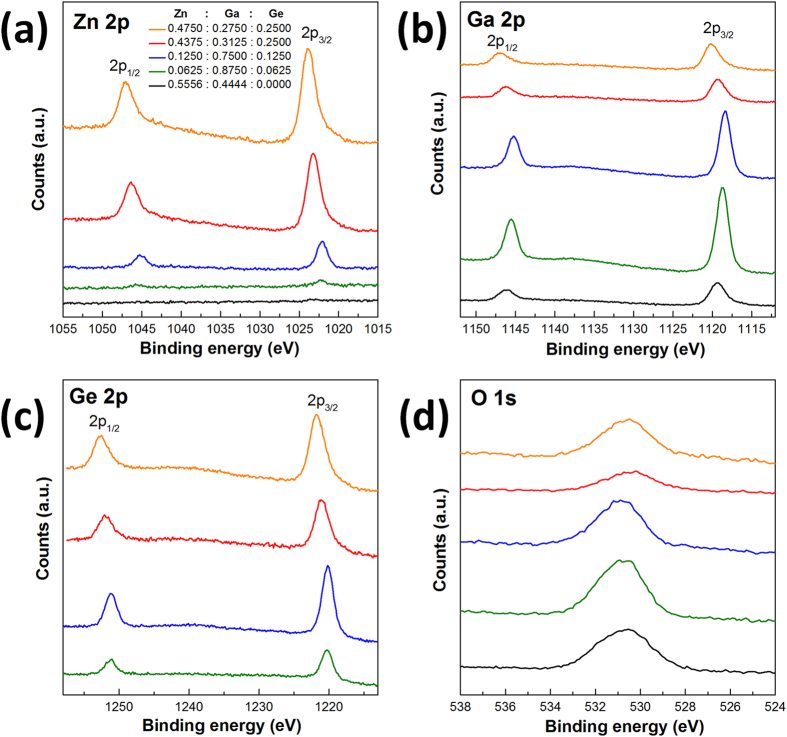
XPS spectra of as-prepared samples: (**a**) Zn 2p peaks, (**b**) Ga 2p peaks, (**c**) Ge 2p peaks and (**d**) O 1s peaks. All peaks are referenced to adventitious carbon C 1 s peak at 284.7 eV.

The surface nature of as-prepared samples was examined by XPS. The narrow-scan of compositional elements Zn, Ga, Ge and O is shown for comparison (Fig. 5). A striking effect of introducing Ge in the structure is elucidated by the increased Zn 2p peaks along with Ge dosage (Fig. 5a). Solid solution (Ga_1-x_Zn_x_)(N_1-x_O_x_) (Entry 3 in Supplementary Table S1) exhibits almost negligible Zn 2p signals whereas a substantially enhanced signal is detected even with adding very small amounts of Ge (starting Zn/Ga/Ge molar ratio = 0.0625/0.8750/0.0625, Entry 28 in Supplementary Table 1S). The effect is quite significant by the fact that much smaller amounts of Zn is used in the raw mixtures. In the light of highly volatile nature of Zn under synthetic conditions here, such monotonic increase of Zn signals along with Ge usage indicates the remarkable stabilization of Zn in the presence of Ge. The large variations in the binding energy of Zn 2p from sample to sample (1022 eV to 1123 eV for Zn 2p_3/2_) suggest changes in the local chemical environment. Similar observation is also noticed in the binding energy of Ga 2p and Ge 2p (Fig. 5b,c) therefore highlights the strong correlation between atomic local environment and starting Zn/Ga/Ge molar ratios. Nevertheless, the binding energy of O 1s all lay around 531 eV, corresponding to the lattice O^2-^ species^39^. The superior Zn stabilizing effect after introducing Ge is also confirmed from the composition analysis. Table 1 listed the surface Zn/Ga/Ge molar ratios for samples that show single wurtizite phase according to XRD analysis. A significant loss of Zn (~93%) can be envisaged from the dramatic decrease of Zn molar ratio after ammonolysis (Entry 3 in Table 1). Therefore, the surface nature of (Ga_1-x_Zn_x_)(N_1-x_O_x_) solid solution is nearly of pure GaN characteristics. On the contrary, considerable amounts of Zn (>60%) are maintained after introducing Ge, especially when the starting Ge molar ratio exceeds 0.2000 in the Zn-Ga-Ge-N-O solid solutions (Entry 7, 11, 12 and 16 in Table 1). It is interesting to note that there is a strong enrichment of Ge at the surface in these samples and might be the reason for Zn stabilization as more Zn^2+^ is needed to balance the Ge^4+^ in the wurtzite structure where cation/anion ratio is equal to one. Bulk atomic composition from EDS analysis also indicate that appreciable amounts of Zn were retained in the structure containing Ge, in contrast to the trivial amounts of Zn left in the (Ga_1-x_Zn_x_)(N_1-x_O_x_) solid solution (Supplementary Fig. S3 and Table S3).

### Theoretical calculations

**Figure 6:**
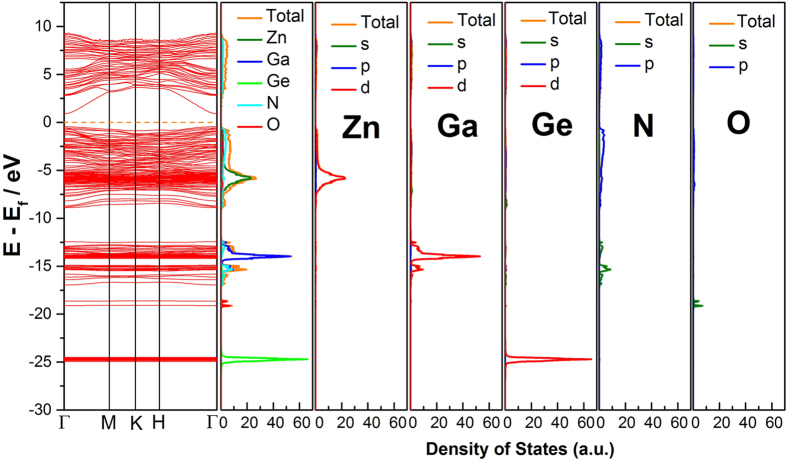
Calculated band structures, total density of states (DOS) and partial density of states (PDOS) of constituent elements for Zn-Ga-Ge-N-O solid solution. Fermi level is denoted by the orange dotted line.

**Figure 7:**
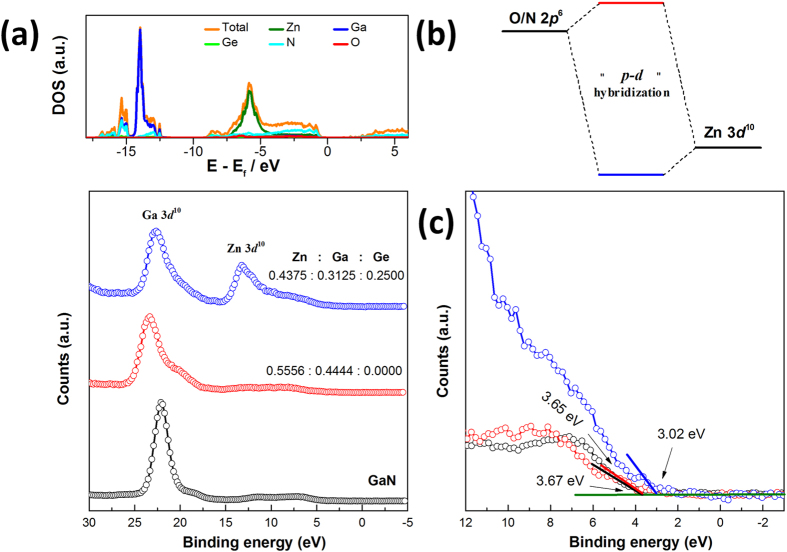
(**a**) calculated density of state versus valence band scan of solid solutions with and without Ge, GaN is showed for comparison, (**b**) schematic representation of Zn 3d and N 2p orbital hybridization, (**c**) valence band maximum (VBM) determination from XPS valence band scan.

In order to elucidate the role of Zn in the quinary Zn-Ga-Ge-N-O solid solutions, we carried out theoretical calculations on their electronic band structures as well as density of states (DOS) close to the Fermi level. The calculated results are presented in Fig. 6 and Supplementary Fig. S5 for enlarged DOS. It is known that Zn plays a critical role in the band gap reduction of (Ga_1-x_Zn_x_)(N_1-x_O_x_) solid solutions^13^. The hybridization between occupied Zn 3d orbitals with O/N 2p orbitals (also called *p-d* repulsion) uplift the valence band maximum (VBM) therefore allows absorption of longer wavelength photons^1^,^40^. This is also the case in Zn-Ga-Ge-N-O solid solutions. The upper part of valence band (VB) is mainly composed of Zn 3d and N 2p orbitals, confirming *p-d* hybridization (Fig. 7a,b). Such hybridization phenomenon occurring exclusively in case of Zn 3d rather than Ga 3d or Ge 3d orbitals can be understood by their individual orbital energy. The relative smaller energy difference between Zn 3d (~−6 eV vs. E_f_) and N 2p (−3 eV vs. E_f_) orbitals favors their hybridization. On the contrary, Ga 3d and Ge 3d orbitals lie deeply at about −15 eV vs. E_f_ and −25 eV vs E_f_, respectively, which prevents their coupling with N 2p orbitals (Fig. 6, PDOS). These orbital interactions can be also evaluated by the width of the band formed. The Ga 3d band and particularly Ge 3d band have much sharper DOS distributions compared to Zn 3d band, indicative of core-level features (Fig. 6). Therefore, the role of Zn in the structure can be understood to raise the VBM in the similar manner of Zn in (Ga_1-x_Zn_x_)(N_1-x_O_x_) solid solution. This is also supported in the experiments of XPS valence band scan (VBS). Figure 7 presents the results for samples with and without Ge. Zn-Ga-Ge-N-O solid solutions typically show two wide bands close to the Fermi level in VBS, which is comparable with calculated DOS (Fig. 7a). These two bands can be assigned to Ga 3d^10^ and Zn 3d^10^ bands, respectively, with the latter being completely absent in the case of (Ga_1-x_Zn_x_)(N_1-x_O_x_) solid solution. These results are consistent with previous analysis that Ge helps to preserve Zn in the structure. Close examination of VBS suggests that the position of VBM is actually controlled by the presence or absence of Zn 3d^10^, approximated 0.6 eV higher VBM being found in solid solution of Zn-Ga-Ge-N-O than (Ga_1-x_Zn_x_)(N_1-x_O_x_) and GaN (Fig. 7c). The nearly equal setting of VBM confirmed the surface nature of (Ga_1-x_Zn_x_)(N_1-x_O_x_) solid solution to be of GaN characteristics. The reason for this is apparent that (Ga_1-x_Zn_x_)(N_1-x_O_x_) solid solution has trivial amounts of Zn at the surface for *p-d* hybridization. Nevertheless, these information is limited to the topmost surface of 1 ~ 3 nm due to the detection limit of XPS techniques^41^. On the other hand, the wurtzite solid solution has wide conduction band (CB) dispersions, implying a high electron mobility of in the CB (Supplementary Fig. S5)^42^,^43^,^44^. The CB is found to have significant contribution from s/p orbitals of Zn, Ga, Ge and N atoms. These orbitals are all of wide distribution so that electrons in these orbitals are essentially delocalized. The calculated band gap 1.33 eV, however, is much smaller compared with experimental value ~2.7 eV which is commonly encountered in calculations using GGA method^45^.

### Photocatalytic properties

**Figure 8:**
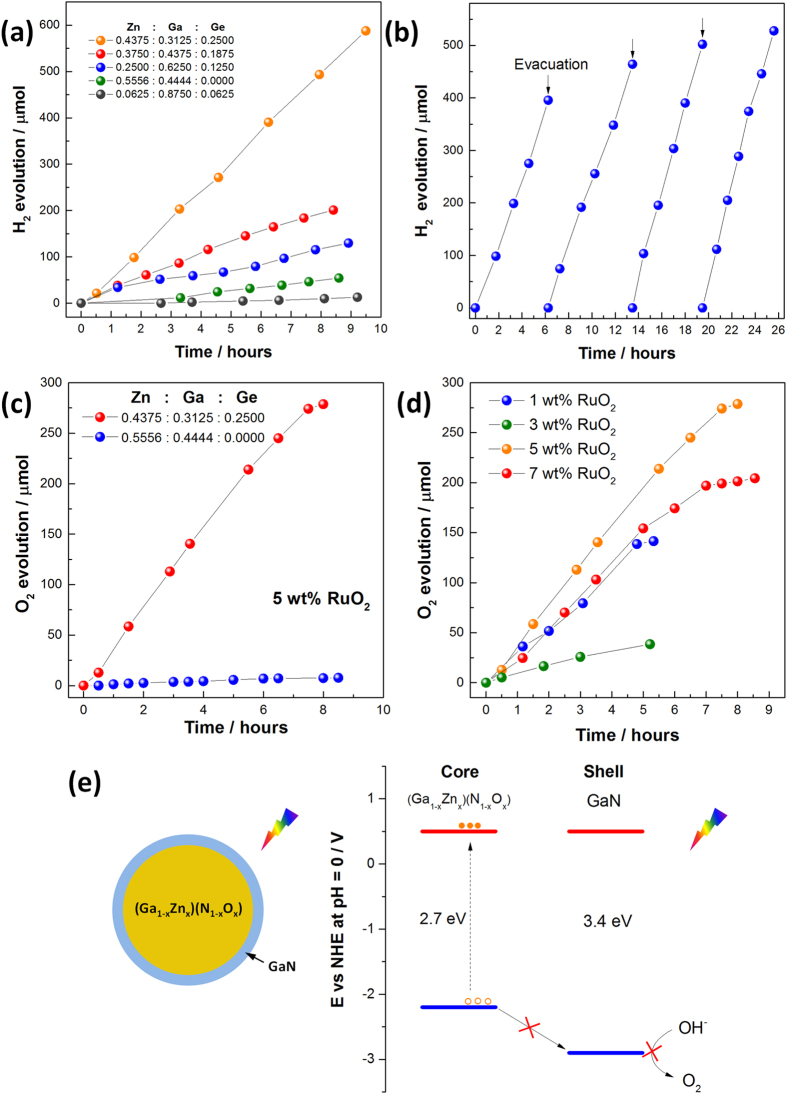
(**a**) Temporal photocatalytic hydrogen production of as-prepared with different starting Zn/Ga/Ge molar ratios, (**b**) repeated time courses of photocatalytic hydrogen production for Zn-Ga-Ge-N-O solid solution (starting Zn/Ga/Ge molar ratio = 0.4375/0.3125/0.2500, Entry 12 in Table 1S). Evacuation was performed at the end of experiments and was labelled by arrow ↓. 1 wt% Pt was used as cocatalyst and oxalic acid (0.025 M, pH = 1.7) was used as hole scavengers. (**c**) Temporal photocatalytic oxygen production of as-prepared Zn-Ga-Ge-N-O solid solution and (Ga_1-x_Zn_x_)(N_1-x_O_x_) solid solution under visible light irradiation (λ ≥ 400), 5 wt% RuO_2_ was used as a cocatalyst. (**d**) Temporal photocatalytic oxygen production of as-prepared Zn-Ga-Ge-N-O solid solution (Entry 12) loaded with different amounts of RuO_2_ cocatalyst, AgNO_3_ (0.005 M) was used as an electron scavenger. (**e**) Schematic representation of internal structure in (Ga_1-x_Zn_x_)(N_1-x_O_x_) solid solution and its photo-oxidation mechanism.

The photocatalytic properties of Zn-Ga-Ge-N-O solid solutions such as hydrogen and oxygen production from water were evaluated using sacrificial reagents under visible light irradiation. Control experiments under dark conditions were firstly performed in order to examine any reactions that do not proceed photocatalytically. No hydrogen or oxygen were detected in the absence of light irradiation thereby exclude any reactions that will lead to hydrogen or oxygen production spontaneously. In the presence of visible light (λ ≥ 400) and oxalic acid aqueous solution, steady state hydrogen production was monitored and the results were plotted in Fig. 8a. The hydrogen production rate of Zn-Ga-Ge-N-O solid solutions was found to be strongly dependent on the starting Zn/Ga/Ge molar ratios. Samples with higher Ge molar ratios generally demonstrate better performance. The highest hydrogen production rate was achieved (~62 μmol/h) for sample with starting Zn/Ga/Ge molar ratio equals to 0.4375/0.3125/0.2500 (Entry 12, Supplementary Table S1). Since this sample has a absorption cut-off at 450 nm, the apparent quantum efficiency approaches as high as 1.01%. Strikingly, such high hydrogen production rate is almost one order of magnitude higher than (Ga_1-x_Zn_x_)(N_1-x_O_x_) solid solution (Entry 3, Supplementary Table S1) under the same conditions (~6.3 μmol/h). Such a poor activity of (Ga_1-x_Zn_x_)(N_1-x_O_x_) solid solution using Pt as a cocatalyst was also reported in the literatures^16^. Surface area is not supposed to play an important role here as samples tested all have a comparable BET surface area at about 4 ~ 6 m^2^/g (Supplementary Table S4). Considering their atomic compositions and microstructures, such high photocatalytic activity is presumably linked to the high Zn content in the structure as well as a good particle crystallinity. Long term testing experiment on Zn-Ga-Ge-N-O solid solution suggests that its photocatalytic hydrogen generation process is quite stable with noticeable improvement after several testing cycles (Fig. 8b). More than 1800 μmol H_2_ was produced within 25 hours, which far exceeds the amounts of catalyst used (600 μmol), confirming a real photocatalytic process.

The photocatalytic O_2_ production experiments were carried out in AgNO_3_ (0.005M) aqueous solution with RuO_2_ as a cocatalyst. At a RuO_2_ loading level of 5 wt%, stable O_2_ evolution was observed in Zn-Ga-Ge-N-O solid solution at a rate of 34.8 μmol/h, corresponding to an apparent quantum efficiency of 1.14%. This rate is more than 36 times higher than (Ga_1-x_Zn_x_)(N_1-x_O_x_) solid solution (~0.95 μmol/h) under the same conditions albeit they have comparable light absorbance. Nevertheless, O_2_ production rate was found to be strongly associated with amounts of RuO_2_ loaded, with 5 wt% being the optimal value (Fig. 8d). Similar observations were also noticed during the investigation of (Ga_1-x_Zn_x_)(N_1-x_O_x_) solid solution^20^. In the light of atomic distributions from bulk to the surface (Table 1, Supplementary Fig. S3 and Table S3), such a poor activity of (Ga_1-x_Zn_x_)(N_1-x_O_x_) solid solution is probably due to the unevenly distribution of Zn cations that forms a core-shell structure. The core can be treated as (Ga_1-x_Zn_x_)(N_1-x_O_x_) solid solution while the shell is essentially of GaN characteristics owing to the severe loss of Zn. The formation of GaN shell has negative effect upon the photocatalytic O_2_ production since internal photogenerated holes cannot migrate across the shell which is energetically prohibited (Fig. 8e). The importance of introducing Ge in the Zn-Ga-Ge-N-O solid solutions can therefore be realized as to preserve migration pathways for holes from approaching reaction sites at the surface.

In summary, quinary wurtzite Zn-Ga-Ge-N-O solid solutions were systematically investigated in this work and were prepared by solid state reactions in flowing ammonia atmosphere at high temperatures. Synthetic conditions such as molar ratio of raw materials, calcining temperatures and time were explored. Single phase formation largely relies on proper molar ratios among Zn/Ga/Ge in the starting mixtures. The highest molar ratio of Ge can be used for single phase formation is 25% and the best calcination conditions are 900 °C for 5 hours. Severe Zn evaporation events occurred during the synthesis absent of Ge owing to the volatile nature of Zn under the reducing atmosphere. Great Zn stabilization effect was realized in the presence of Ge and was probably associated with a strong Ge enrichment phenomenon at the surface of these quinary solid solutions. Apart from Zn preservation, particle crystallization was significantly promoted in the presence of Ge and might be linked to the facile growth of *β*-Ge_3_N_4_. Visible light absorption is achieved in all quinary solid solutions and is comparable with (Ga_1-x_Zn_x_)(N_1-x_O_x_) solid solution reported. Theoretical calculation pointed out that the enhanced light absorption is due to the orbital hybridization between Zn 3d and O/N 2p that uplifts valence band maximum (*p-d* repulsion). The lack of Zn at the surface of (Ga_1-x_Zn_x_)(N_1-x_O_x_) solid solution therefore renders its surface GaN characteristics. Photocatalytic H_2_ production was achieved in these quinary solid solutions under visible light irradiation and its production rate was inherently correlated with Zn content as well as particle crystallinity. Much enhanced photocatalytic O_2_ production rate was observed in quinary solid solutions than (Ga_1-x_Zn_x_)(N_1-x_O_x_) and was probably due to the absence of GaN shell formed after Zn evaporation that prevents efficient hole migration to the surface. Apparent quantum efficiency for optimized samples approaches 1.01% for hydrogen production and 1.14% for oxygen production.

## Methods

### Material synthesis

Zn-Ga-Ge-N-O solid solutions were prepared by calcining the mixtures of ZnO (Aladdin, 99.9%), Ga_2_O_3_ (Aladdin, 99.99%) and GeO_2_ (Aladdin, 99.999%) powders in a tube furnace under flowing ammonia at high temperatures. Different molar ratios between Zn, Ga and Ge, and different calcining temperatures and time were used in order to evaluate the factors that control the physiochemical properties of Zn-Ga-Ge-N-O solid solutions (Supplementary Table S1). (Ga_1-x_Zn_x_)(N_1-x_O_x_) solid solution, GaN and *β*-Ge_3_N_4_ were prepared under the same conditions for comparison. In a typical synthesis, 1.4388 g ZnO, 1.1716 g Ga_2_O_3_ and 1.0464 g GeO_2_ were thoroughly blend using an agate mortar and pestle for 30 min. The mixtures were then transferred into an alumina boat and were heated in a tube furnace at 900 °C for 5 hours. Pure ammonia (Jiaya Chemicals, 99.999%) was used as a carrier gas with a flow rate approximately 250 ml/min.

### Materials characterization

Phase purity was examined by using X-ray powder diffraction (XRD) techniques (Bruker D8 Focus diffractometer). Incident radiation used were Cu K_α1_ (λ = 1.5406 Å) and Cu K_α2_ (λ = 1.5444 Å). The step size for data collection was 0.01° with a collection time 100 s for each step. General Structure Analysis System (GSAS) software package was applied to perform Rietveld refinement^46^. Microstructures and the bulk composition of prepared samples were analyzed by a field emission scanning electron microscope (Hitachi S4800) and transmission electron microscope (JEM 2100F) equipped with a Mica energy dispersive X-ray spectroscopy (EDS) analysis system. Surface compositions of prepared samples and their valence band were analyzed using X-ray photoelectron spectroscopy (Thermo Escalab 250, a monochromatic Al Kα X-ray source). All binding energies were referenced to the C 1s peak (284.7 eV) arising from adventitious carbon^41^. Diffuse reflectance spectra were collected and analyzed using a UV-Vis spectrophotometer (JASCO-750) and JASCO software suite. BaSO_4_ was used as a reference non-absorbing material. The Raman spectra of the prepared sample were performed on a Thermal Scientific DXR Raman spectrometer equipped with visible (633 nm) laser excitation (He-Ne laser) and used a confocal microscope for focusing the laser beam onto the sample. Spectral resolution is around 2 cm^−1^. Surface areas were analyzed on a Micro-meritics instrument TriStar 3000 and were calculated via the Brunauer-Emmett-Teller (BET) model.

### Photocatalytic activity

Photocatalytic activity of as-prepared samples was evaluated in a top-irradiation-type reactor connected to a gas-closed circulation and evacuation system (Perfect Light, Labsolar-IIIAG). In a typical experiment, 0.1 g sample powders were dispersed in 100 ml aqueous solution, which was sealed in the reactor. Oxalic acid (0.025 M) and AgNO_3_ (0.005M) were used as sacrificial agents for photoreduction and photooxidation reactions, respectively^3^,^47^,^48^. Pt was used as a cocatalyst for photocatalytic hydrogen production and was loaded according to previous reports^47^: H_2_PtCl_6_ aqueous solution was impregnated into sample powders and was heated on a hot-plate at 90 °C until dry. Thereafter the temperature was raised to 180 °C for 2 hours to fully decompose H_2_PtCl_6_ into Pt nanoparticles. RuO_2_ was used as a cocatalytst for photocatalytic oxygen production and was loaded following the procedures in the literatures^20^: ethyl acetone ruthenium dissolved in tetrahydrofuran (THF) solution was impregnated into sample powders in a water bath at 80 °C. The temperature was then raised to 350 °C for 2 hours for the conversion of ruthenium complex species into RuO_2_. A 300 W Xenon lamp (Perfect Light, PLX-SXE300) was used as a light source which is coupled with a UV cut-off filter (λ ≥ 400 nm) to generate visible light irradiation. The photon flux of the lamp is calibrated using a quantum meter (Apogee MP-300). The recorded photon flux is ~879.31 μmol/m^2^/s for visible light irradiation (400 nm ≤ λ ≤ 450 nm).Water jacket was used to stabilize reactor temperature around 20 °C. The gas component within the reactor was then analyzed using an on-lined gas chromatograph (TECHCOMP, GC7900) with a TCD detector (5 Å molecular sieve columns and N_2_ carrier).

### Theoretical calculations

Theoretical calculations were performed using the density functional theory (DFT) implemented in the Vienna ab initio simulation package (VASP)^49^. Perdew, Burke and Ernzerhof (PBE) exchange-correclation functional within the generalized gradient approximation^50^ and the projector augmented-wave pseudopotential were applied^51^. A 2 × 2 × 1 super cell of wurtzite GaN (a = b = 6.38 Å, c = 10.37 Å, α = β = 90 ° and γ = 120 °) with hexagonal symmetry was constructed for simulations of quinary Zn-Ga-Ge-N-O solid solution (total atom number = 32). The structure was considered by assuming that 10 Ga atoms out of 16 were substituted by 6 Zn atoms and 4 Ge at the cation site and 2 N atoms out of 16 were replaced by 2 O atoms at the anion site, respectively. Detailed structural information can be found in the supplementary information. All geometry structures were fully relaxed until the forces on each atom are less than 0.01 eV/Å. Static calculations were done with a 4 × 4 × 3 Monkhorst-Pack k-point grid^52^.

## Additional Information

**How to cite this article**: Xie, Y. *et al.* Quinary wurtzite Zn-Ga-Ge-N-O solid solutions and their photocatalytic properties under visible light irradiation. *Sci. Rep.* **6**, 19060; doi: 10.1038/srep19060 (2016).

## Supplementary Material

Supplementary Information

## Figures and Tables

**Table 1:** Surface Zn/Ga/Ge molar ratios after ammonolysis for samples with different starting Zn/Ga/Ge molar ratios according to XPS analysis.

Entry	Starting Zn/Ga/Ge molar ratio before ammonolysis			Surface Zn/Ga/Ge molar ratios after ammonolysis from XPS		
	Zn	Ga	Ge	Zn	Ga	Ge
3	0.5556	0.4444	0.0000	0.0400	0.9600	0.0000
7	0.4750	0.2750	0.2500	0.2830	0.1987	0.5184
8	0.4750	0.2750	0.2500	0.2672	0.3138	0.4190
11	0.4375	0.3125	0.2500	0.3081	0.2258	0.4661
12	0.4375	0.3125	0.2500	0.2634	0.3385	0.3982
16	0.4000	0.3500	0.2500	0.2897	0.2894	0.4209
17	0.3750	0.4375	0.1875	0.1842	0.4638	0.3521
19	0.3300	0.5000	0.1700	0.1404	0.5026	0.3569
20	0.3300	0.4700	0.2000	0.1682	0.4880	0.3439
23	0.3000	0.5000	0.2000	0.1420	0.4650	0.3930
24	0.2500	0.6250	0.1250	0.0938	0.6719	0.2343
26	0.1250	0.7500	0.1250	0.0571	0.6016	0.3413
28	0.0625	0.8750	0.0625	0.0184	0.7818	0.1998
29	0.0000	1.0000	0.0000	0.0000	1.0000	0.0000
30	0.0000	0.0000	1.0000	0.0000	0.0000	1.0000

Samples are numbered following the sequence in Table 1 S and only those containing single wurtzite phase are considered.

## References

[b1] Maeda K. *et al.* Photocatalyst releasing hydrogen from water—Enhancing catalytic performance holds promise for hydrogen production by water splitting in sunlight. Nature 440, 295–295, 10.1038/440295a (2006). 16541063

[b2] Lee Y. *et al.* Zinc germanium oxynitride as a photocatalyst for overall water splitting under visible light. J. Phys. Chem. C 111, 1042–1048, 10.1021/Jp0656532 (2007).

[b3] Xu X. X., Randorn C., Efstathiou P. & Irvine J. T. S. A red metallic oxide photocatalyst. Nat. Mater. 11, 595–598, 10.1038/Nmat3312 (2012). 22543300

[b4] Zou Z. G., Ye J. H., Sayama K. & Arakawa H. Direct splitting of water under visible light irradiation with an oxide semiconductor photocatalyst. Nature 414, 625–627 (2001). 11740556 10.1038/414625a

[b5] Kudo A. Z-scheme photocatalyst systems for water splitting under visible light irradiation. Mrs Bull. 36, 32–38, 10.1557/Mrs.2010.3 (2011).

[b6] Kato H., Asakura K. & Kudo A. Highly efficient water splitting into H-2 and O-2 over lanthanum-doped NaTaO3 photocatalysts with high crystallinity and surface nanostructure. J. Am. Chem. Soc. 125, 3082–3089, 10.1021/Ja027751g (2003). 12617675

[b7] Sun X. Q. *et al.* Photocatalytic hydrogen production over chromium doped layered perovskite Sr2TiO4. Inorg. Chem. 54, 7445–7453, 10.1021/acs.inorgchem.5b01042 (2015). 26171625

[b8] Lv M. L. *et al.* Bismuth and Chromium co-doped strontium titanates and their photocatalytic properties under visible light irradiation. Phys. Chem. Chem. Phys., 10.1039/c5cp03889h (2015). 26387833

[b9] Wu F. F. *et al.* Efficient photocatalytic oxygen production over nitrogen doped Sr4Nb2O9 under visible light irradiation. ChemCatChem, 10.1002/cctc.201501035R1 (2015).

[b10] Chen H. M. *et al.* Efficient charge separation based on type-II g-C3N4/TiO2-B nanowire/tube heterostructure photocatalysts. Dalton T. 44, 13030–13039, 10.1039/c5dt01757b (2015). 26102218

[b11] Sato J. *et al.* Photocatalytic activity for water decomposition of RuO2-dispersed Zn2GeO4 with d(10) configuration. J. Phys. Chem. B 108, 4369–4375, 10.1021/Jp0373189 (2004). 16853996

[b12] Lee Y. G., Teramura K., Hara M. & Domen K. Modification of (Zn1 + xGe)(N2Ox) solid solution as a visible light driven photocatalyst for overall water splitting. Chem. Mater. 19, 2120–2127, 10.1021/Cm062980d (2007).

[b13] Maeda K. & Domen K. Solid Solution of GaN and ZnO as a Stable Photocatalyst for Overall Water Splitting under Visible Light. Chem. Mater. 22, 612–623, 10.1021/Cm901917a (2010).

[b14] Liu J. *et al.* Metal-free efficient photocatalyst for stable visible water splitting via a two-electron pathway. Science 347, 970–974, 10.1126/science.aaa3145 (2015). 25722405

[b15] Ohno T., Bai L., Hisatomi T., Maeda K. & Domen K. Photocatalytic Water Splitting Using Modified GaN:ZnO Solid Solution under Visible Light: Long-Time Operation and Regeneration of Activity. J. Am. Chem. Soc. 134, 8254–8259, 10.1021/Ja302479f (2012). 22524238

[b16] Maeda K., Teramura K. & Domen K. Development of cocatalysts for photocatalytic overall water splitting on (Ga1-xZnx)(N1-xOx) solid solution. Catal. Surv. Asia 11, 145–157, 10.1007/s10563-007-9032-2 (2007).

[b17] Adeli B. & Taghipour F. A Review of Synthesis Techniques for Gallium-Zinc Oxynitride Solar-Activated Photocatalyst for Water Splitting. ECS J. Solid State Sc. 2, Q118–Q126, 10.1149/2.022307jss (2013).

[b18] Maeda K. *et al.* GaN : ZnO solid solution as a photocatalyst for visible-light-driven overall water splitting. J. Am. Chem. Soc. 127, 8286–8287, 10.1021/Ja0518777 (2005). 15941253

[b19] Hisatomi T., Maeda K., Takanabe K., Kubota J. & Domen K. Aspects of the Water Splitting Mechanism on (Ga1-xZnx)(N1-xOx) Photocatalyst Modified with Rh2-yCryO3 Cocatalyst. J. Phys. Chem. C 113, 21458–21466, 10.1021/Jp9079662 (2009).

[b20] Teramura K. *et al.* Characterization of ruthenium oxide nanocluster as a cocatalyst with (Ga1-xZnx)(N1-xOx) for photocatalytic overall water splitting. J. Phys. Chem. B 109, 21915–21921, 10.1021/Jp054313y (2005). 16853847

[b21] Maeda K. *et al.* Overall water splitting on (Ga1-xZnx)(N1-xOx) solid solution photocatalyst: Relationship between physical properties and photocatalytic activity. J. Phys. Chem. B 109, 20504–20510, 10.1021/Jp053499y (2005). 16853653

[b22] Yoshida M. *et al.* Photoluminescence Spectroscopic and Computational Investigation of the Origin of the Visible Light Response of (Ga1-xZnx)(N1-xOx) Photocatalyst for Overall Water Splitting. J. Phys. Chem. C 114, 15510–15515, 10.1021/Jp100106y (2010).

[b23] Maeda K., Teramura K. & Domen K. Effect of post-calcination on photocatalytic activity of (Ga1-xZnx)(N1-xOx) solid solution for overall water splitting under visible light. J. Catal. 254, 198–204, 10.1016/j.jcat.2007.12.009 (2008).

[b24] Maeda K. *et al.* Preparation of Core-Shell-Structured Nanoparticles (with a Noble-Metal or Metal Oxide Core and a Chromia Shell) and Their Application in Water Splitting by Means of Visible Light. Chem-Eur. J. 16, 7750–7759, 10.1002/chem.201000616 (2010). 20564294

[b25] Isogai S. *et al.* Composite of Rh (y) Cr2-y O-3/(Ga1-x Zn (x) )(N1-x O (x) ) Photocatalysts with Hydrophobic Polytetrafluoroethylene (PTFE) Membranes for the Fabrication of Novel Reaction Sites for Water Vapor Splitting Under Visible Light. Catal. Lett. 143, 150–153, 10.1007/s10562-012-0950-x (2013).

[b26] Maeda K. *et al.* Characterization of Rh-Cr mixed-oxide nanoparticles dispersed on (Ga1-xZnx)(N1-xOx) as a cocatalyst for visible-light-driven overall water splitting. J. Phys. Chem. B 110, 13753–13758, 10.1021/Jp061829o (2006). 16836320

[b27] Maeda K. *et al.* Roles of Rh/Cr2O3 (core/shell) nanoparticles photodeposited on visible-light-responsive (Ga1-xZnx)(N1-xOx) solid solutions in photocatalytic overall water splitting. J. Phys. Chem. C 111, 7554–7560, 10.1021/Jp071056j (2007).

[b28] Maeda K., Hashiguchi H., Masuda H., Abe R. & Domen K. Photocatalytic activity of (Ga1-xZnx)(N1-xOx) for visible-light-driven H-2 and O-2 evolution in the presence of sacrificial reagents. J. Phys. Chem. C 112, 3447–3452, 10.1021/Jp710758q (2008).

[b29] McDermott E. J. *et al.* Structural and Band Gap Investigation of GaN:ZnO Heterojunction Solid Solution Photocatalyst Probed by Soft X-ray Spectroscopy. J. Phys. Chem. C 116, 7694–7700, 10.1021/Jp301231p (2012).

[b30] Yashima M., Yamada H., Maeda K. & Domen K. Experimental visualization of covalent bonds and structural disorder in a gallium zinc oxynitride photocatalyst (Ga1-xZnx)(N1-xOx): origin of visible light absorption. Chem. Commun. 46, 2379–2381, 10.1039/B922008a (2010). 20309458

[b31] Yang M. H., MacLeod M. J., Tessier F. & DiSalvo F. J. Mesoporous Metal Nitride Materials Prepared from Bulk Oxides. J. Am. Ceram. Soc. 95, 3084–3089, 10.1111/j.1551-2916.2012.05351.x (2012).

[b32] Hirai T. *et al.* Origin of visible light absorption in GaN-Rich (Ga1-xZnx)(N1-xOx) photocatalysts. J. Phys. Chem. C 111, 18853–18855, 10.1021/Jp709811k (2007).

[b33] Shannon R. D. & Prewitt C. T. Effective Ionic Radii in Oxides and Fluorides. Acta Crystallographica B25, 925–946 (1969).

[b34] Butler J. E. & Oleynik I. A mechanism for crystal twinning in the growth of diamond by chemical vapour deposition. Philos T R Soc A 366, 295–310, 10.1098/rsta.2007.2152 (2008). 18024361

[b35] Lide D. R. CRC handbook of chemistry and physics, 89th edition. 2736 (CRC Press, 2008).

[b36] Maeda K., Teramura K., Saito N., Inoue Y. & Domen K. Photocatalytic overall water splitting on gallium nitride powder. B. Chem. Soc. Jpn. 80, 1004–1010, 10.1246/Bcsj.80.1004 (2007).

[b37] Maeda K., Saito N., Lu D. L., Inoue Y. & Domen K. Photocatalytic properties of RuO2-loaded beta-Ge3N4 for overall water splitting. J. Phys. Chem. C 111, 4749–4755, 10.1021/Jp067254c (2007).

[b38] Sato J. *et al.* RuO2-loaded beta-Ge3N4 as a non-oxide photocatalyst for overall water splitting. J. Am. Chem. Soc. 127, 4150–4151, 10.1021/Ja042973v (2005). 15783179

[b39] Battistoni C., Dormann J. L., Fiorani D., Epaparazzo E. & Viticoli S. An XPS and Mossbauer study of the electronic properties of ZnCrxGa2-xO4 spinel solid solution. Solid State Commun. 39, 581–585 (1981).

[b40] Wei S. H. & Zunger A. Role of metal d states in II-VI semiconductors. Phys. Rev. B 37, 8958–8981 (1988). 10.1103/physrevb.37.8958 9944267

[b41] Van der Heide P. X-ray photoelectron Spectroscopy—An introduction to principles and practices. (John Wiley & Sons, Inc, 2012).

[b42] Cox P. A. The electronic structure and chemistry of solids. 259 (Oxford University Press, 1987).

[b43] Cox P. A. Transition metal oxides: An introduction to their electronic structure and properties. (Oxford university press, 1992).

[b44] Kittel C. Introduction to solid state physics. (Wiley, 1953).

[b45] Xiao H., Tahir-Kheli J. & Goddard W. A. Accurate Band Gaps for Semiconductors from Density Functional Theory. J. Phys. Chem. Lett. 2, 212–217, 10.1021/Jz101565j (2011).

[b46] Larson A. C. & Von Dreele R. B. GSAS-Generalised Crystal Structure Analysis System. Los Alamos National Laboratory Report No. LA-UR-86-748 (1994).

[b47] Xu X. X., Liu G., Randorn C. & Irvine J. T. S. g-C(3)N(4) coated SrTiO(3) as an efficient photocatalyst for H(2) production in aqueous solution under visible light irradiation. Int. J. Hydrogen Energ. 36, 13501–13507, 10.1016/j.ijhydene.2011.08.052 (2011).

[b48] Xu X. X., Liu G. & Azad A. K. Visible light photocatalysis by *in situ* growth of plasmonic Ag nanoparticles upon AgTaO3. Int. J. Hydrogen Energ. 40, 3672–3678, (2015).

[b49] Kresse G. & Furthmuller J. Efficient iterative schemes for ab initio total-energy calculations using a plane-wave basis set. Phys. Rev. B 54, 11169–11186 (1996). 10.1103/physrevb.54.11169 9984901

[b50] Perdew J. P., Burke K. & Ernzerhof M. Generalized gradient approximation made simple. Phys. Rev. Lett. 77, 3865–3868 (1996). 10062328 10.1103/PhysRevLett.77.3865

[b51] Kresse G. & Joubert D. From ultrasoft pseudopotentials to the projector augmented-wave method. Phys. Rev. B 59, 1758–1775 (1999).

[b52] Monkhorst H. J. & Pack J. D. Special Points for Brillouin-Zone Integrations. Phys. Rev. B 13, 5188–5192 (1976).

