# Supplementary material for: Quinary wurtzite Zn-Ga-Ge-N-O solid solutions and their photocatalytic properties under visible light irradiation
Source: Sci Rep. 2016 Jan 12;6:19060. doi: 10.1038/srep19060 (PMC4709556; doi:10.1038/srep19060)
Supplement: Supplementary Information [file srep19060-s1.doc]

**Supplementary Information**

**Quinary wurtzite Zn-Ga-Ge-N-O solid solutions and their photocatalytic properties under visible light irradiation**

**Yinghao Xie^a^, Fangfang Wu^a^, Xiaoqin Sun^a^, Hongmei Chen^a^, Meilin Lv^a^, Shuang Ni^b^, Gang Liu^c^ and Xiaoxiang Xu^a^,***

*^a^Shanghai Key Lab of Chemical Assessment and Sustainability, Department of Chemistry, Tongji University, 1239 Siping Road, Shanghai, 200092, China*

*Email:* [*xxxu@tongji.edu.cn*](mailto:xxxu@tongji.edu.cn)*, telephone: +86-21-65986919*

*^b^Science and Technology on Plasma Physics Laboratory, Laser Fusion Research Center, China Academy of Engineering Physics, Mianyang 621900, China*

*^c^Shenyang National laboratory for Materials Science, Institute of Metal Research, Chinese Academy of Science, 72 Wenhua Road, Shenyang 110016, China*

Table of Contents

**Figure S1 Rietveld refinement plot of XRD patterns for product with starting Zn/Ga/Ge molar ratio (0.4375: 0.3125: 0.2500) (Entry 12 in Table 1S). Nitridation temperature and time is 900 °C and 5 hours. Goodness-of-fit parameters are *R_p_* = 6.53%, *R_wp_* = 8.38 and χ^2^ = 2.808. The refined crystal structure is displayed as inserted image.**

**Figure S2 (a) TEM and (b) HRTEM images of product with starting Zn/Ga/Ge molar ratio (0.4375: 0.3125: 0.2500) (Entry 12 in Table 1S); (c) TEM and (d) HRTEM images of (Ga_1-x_Zn_x_)(N_1-x_O_x_) solid solution with starting Zn/Ga molar ratio (0.5556: 0.4444)( Entry 3 in Table 1S).**

**Figure S3 Selected area electron dispersive X-ray spectroscopy analysis on product with starting Zn/Ga/Ge molar ratio (0.4375: 0.3125: 0.2500) (Entry 12 in Table 1S) (a) and (Ga_1-x_Zn_x_)(N_1-x_O_x_) solid solution with starting Zn/Ga molar ratio (0.5556: 0.4444)( Entry 3 in Table 1S) (b); atomic compositions at selected area marked in TEM images were listed in the table inserted in the spectra.**

**Figure S4 Simulated 2×2×1 wurtzite super cell used in DFT calculations for Zn-Ga-Ge-N-O solid solution (Zn_6_Ga_6_Ge_4_N_14_O_2_)**

**Figure S5 Calculated total density of states (DOS) and partial density of states (PDOS) of constituent elements around Fermi level, images are enlarged from Figure 8 for clarity**

**Table S1 Detailed synthetic conditions for samples investigated and the resultant phase compositions**

**Table S2 Structural information for samples containing single wurtzite phase from Table S1**

**Table S3 Bulk atomic composition from EDS analysis for samples containing single wurtzite phase from Table S1**

**Table S4 BET surface area of selected samples containing single wurtzite phase**

Fig. S1 Rietveld refinement plot of XRD patterns for product with starting Zn/Ga/Ge molar ratio (0.4375: 0.3125: 0.2500) (**Entry 12** in Table 1S). Nitridation temperature and time is 900 °C and 5 hours. Goodness-of-fit parameters are *R_p_* = 6.53%, *R_wp_* = 8.38 and χ^2^ = 2.808. The refined crystal structure is displayed as inserted image.

Fig. S2 (a) TEM and (b) HRTEM images of product with starting Zn/Ga/Ge molar ratio (0.4375: 0.3125: 0.2500) (**Entry 12** in Table 1S); (c) TEM and (d) HRTEM images of (Ga_1-x_Zn_x_)(N_1-x_O_x_) solid solution with starting Zn/Ga molar ratio (0.5556: 0.4444)( **Entry 3** in Table 1S).

Fig. S3 Selected area electron dispersive X-ray spectroscopy analysis on product with starting Zn/Ga/Ge molar ratio (0.4375: 0.3125: 0.2500) (**Entry 12** in Table 1S) (a) and (Ga_1-x_Zn_x_)(N_1-x_O_x_) solid solution with starting Zn/Ga molar ratio (0.5556: 0.4444)( **Entry 3** in Table 1S) (b); atomic compositions at selected area marked in TEM images were listed in the table inserted in the spectra.

Fig. S4 Simulated 2×2×1 wurtzite super cell used in DFT calculations for Zn-Ga-Ge-N-O solid solution (Zn_6_Ga_6_Ge_4_N_14_O_2_)

Fig. S5 Calculated total density of states (DOS) and partial density of states (PDOS) of constituent elements around Fermi level, images are enlarged from Figure 8 for clarity.

Table S1 Detailed synthetic conditions for samples investigated and the resultant phase compositions

| Entry | Cation molar ratio before nitridation | | | Nitridation temperature | Nitridation time | Phase composition |
| --- | --- | --- | --- | --- | --- | --- |
|  | Zn | Ga | Ge |  |  |  |
| **1** | 0.8333 | 0.0000 | 0.1667 | 900°C | 5h | ZnGeN_2_ + Ge_3_N_4_ |
| **2** |  |  |  | 900°C | 10h | ZnGeN_2_ + Ge_3_N_4_ |
| **3** | 0.5556 | 0.4444 | 0.0000 | 900°C | 5h | Single wurtzite |
| **4** | 0.5000 | 0.3300 | 0.1700 | 900°C | 5h | Two wurtzites |
| **5** | 0.5000 | 0.2500 | 0.2500 | 900°C | 5h | Two wurtzites |
| **6** | 0.4750 | 0.2750 | 0.2500 | 850°C | 5h | Two wurtzites |
| **7** |  |  |  | 900°C | 3h | Single wurtzite |
| **8** |  |  |  | 900°C | 5h | Single wurtzite |
| **9** | 0.4375 | 0.2500 | 0.3125 | 900°C | 5h | Two wurtzites + Ge_3_N_4_ |
| **10** | 0.4375 | 0.3125 | 0.2500 | 850°C | 5h | Two wurtzites |
| **11** |  |  |  | 900°C | 3h | Single wurtzite |
| **12** |  |  |  | 900°C | 5h | Single wurtzite |
| **13** |  |  |  | 900°C | 10h | Wurtzite + Ge_3_N_4_ |
| **14** | 0.4000 | 0.3500 | 0.2500 | 850°C | 5h | Wurtzite + Ge_3_N_4_ + Ga_2_O_3_ |
| **15** |  |  |  | 900°C | 3h | Wurtzite + Ge_3_N_4_ + Ga_2_O_3_ |
| **16** |  |  |  | 900°C | 5h | Single wurtzite |
| **17** | 0.3750 | 0.4375 | 0.1875 | 900°C | 5h | Single wurtzite |
| **18** | 0.3750 | 0.3125 | 0.3125 | 900°C | 5h | Wurtzite + Ge_3_N_4_ |
| **19** | 0.3300 | 0.5000 | 0.1700 | 900°C | 5h | Single wurtzite |
| **20** | 0.3300 | 0.4700 | 0.2000 | 900°C | 5h | Single wurtzite |
| **21** | 0.3300 | 0.4200 | 0.2500 | 900°C | 5h | Wurtzite + Ge_3_N_4_ |
| **22** | 0.3300 | 0.3300 | 0.3300 | 900°C | 5h | Wurtzite + Ge_3_N_4_ |
| **23** | 0.3000 | 0.5000 | 0.2000 | 900°C | 5h | Single wurtzite |
| **24** | 0.2500 | 0.6250 | 0.1250 | 900°C | 5h | Single wurtzite |
| **25** | 0.2500 | 0.5000 | 0.2500 | 900°C | 5h | Wurtzite + Ge_3_N_4_ |
| **26** | 0.1250 | 0.7500 | 0.1250 | 900°C | 5h | Single wurtzite |
| **27** | 0.1250 | 0.6250 | 0.2500 | 900°C | 5h | Wurtzite + Ge_3_N_4_ |
| **28** | 0.0625 | 0.8750 | 0.0625 | 900°C | 5h | Single wurtzite |
| **29** | 0.0000 | 1.0000 | 0.0000 | 900°C | 5h | GaN |
| **30** | 0.0000 | 0.0000 | 1.0000 | 900°C | 5h | *β*-Ge_3_N_4_ |

Table S2 Structural information for samples containing single wurtzite phase from Table S1

| Entry | Cation molar ratio before nitridation | | | Space group | Unit cell parameters calculated from Rietveld refinement | | |
| --- | --- | --- | --- | --- | --- | --- | --- |
|  | Zn | Ga | Ge |  | *a* / Å | *c* / Å | *V* / Å^3^ |
| **3** | 0.5556 | 0.4444 | 0.0000 | *P* 6_3_*mc* | 3.1945(1) | 5.1896(2) | 45.866(4) |
| **7** | 0.4750 | 0.2750 | 0.2500 | *P* 6_3_*mc* | 3.1943(2) | 5.1917(3) | 45.879(6) |
| **8** | 0.4750 | 0.2750 | 0.2500 | *P* 6_3_*mc* | 3.1912(2) | 5.1880(3) | 45.756(5) |
| **11** | 0.4375 | 0.3125 | 0.2500 | *P* 6_3_*mc* | 3.1935(3) | 5.1902(4) | 45.843(8) |
| **12** | 0.4375 | 0.3125 | 0.2500 | *P* 6_3_*mc* | 3.1901(1) | 5.1855(2) | 45.705(3) |
| **16** | 0.4000 | 0.3500 | 0.2500 | *P* 6_3_*mc* | 3.1935(2) | 5.1888(3) | 45.829(4) |
| **17** | 0.3750 | 0.4375 | 0.1875 | *P* 6_3_*mc* | 3.1972(2) | 5.1896(4) | 45.943(7) |
| **19** | 0.3300 | 0.5000 | 0.1700 | *P* 6_3_*mc* | 3.1976(2) | 5.1881(4) | 45.940(7) |
| **20** | 0.3300 | 0.4700 | 0.2000 | *P* 6_3_*mc* | 3.1954(2) | 5.1883(3) | 45.879(5) |
| **23** | 0.3000 | 0.5000 | 0.2000 | *P* 6_3_*mc* | 3.1948(2) | 5.1867(3) | 45.847(5) |
| **24** | 0.2500 | 0.6250 | 0.1250 | *P* 6_3_*mc* | 3.1956(3) | 5.1902(4) | 45.900(7) |
| **26** | 0.1250 | 0.7500 | 0.1250 | *P* 6_3_*mc* | 3.1917(3) | 5.1877(4) | 45.769(8) |
| **28** | 0.0625 | 0.8750 | 0.0625 | *P* 6_3_*mc* | 3.1900(2) | 5.1806(3) | 45.656(4) |
| **29** | 0.0000 | 1.0000 | 0.0000 | *P* 6_3_*mc* | 3.1908(5) | 5.1870(8) | 45.74(2) |
| **30** | 1.0000 | 0.0000 | 0.0000 | *P* 6_3_*mc* | 3.2498(1) | 5.2063(2) | 47.619(3) |

Table S3 Bulk atomic composition from EDS analysis for samples containing single wurtzite phase from Table S1

| Entry | Starting Zn/Ga/Ge molar ratios before nitridation | | | Bulk atomic percentage after nitridation / 100% | | | | |
| --- | --- | --- | --- | --- | --- | --- | --- | --- |
|  | Zn | Ga | Ge | Zn | Ga | Ge | N | O |
| **3** | 0.5556 | 0.4444 | 0.0000 | 2.85 | 41.10 | 0.00 | 48.06 | 7.98 |
| **7** | 0.4750 | 0.2750 | 0.2500 | 15.55 | 17.43 | 13.14 | 46.51 | 7.36 |
| **8** | 0.4750 | 0.2750 | 0.2500 | 13.98 | 18.68 | 12.19 | 47.71 | 7.45 |
| **11** | 0.4375 | 0.3125 | 0.2500 | 20.91 | 26.99 | 9.90 | 35.40 | 6.80 |
| **12** | 0.4375 | 0.3125 | 0.2500 | 15.90 | 17.85 | 11.98 | 44.41 | 9.85 |
| **16** | 0.4000 | 0.3500 | 0.2500 | 12.71 | 17.62 | 11.83 | 51.10 | 6.73 |
| **17** | 0.3750 | 0.4375 | 0.1875 | 11.65 | 20.73 | 8.74 | 46.07 | 12.81 |
| **19** | 0.3300 | 0.5000 | 0.1700 | 10.13 | 21.88 | 7.28 | 48.40 | 12.32 |
| **20** | 0.3300 | 0.4700 | 0.2000 | 8.78 | 16.84 | 9.17 | 48.78 | 16.43 |
| **23** | 0.3000 | 0.5000 | 0.2000 | 11.55 | 26.36 | 8.79 | 42.47 | 10.82 |
| **24** | 0.2500 | 0.6250 | 0.1250 | 9.00 | 29.73 | 5.71 | 45.49 | 10.08 |
| **26** | 0.1250 | 0.7500 | 0.1250 | 5.92 | 35.09 | 4.86 | 45.40 | 8.73 |
| **28** | 0.0625 | 0.8750 | 0.0625 | 2.79 | 34.79 | 2.92 | 47.96 | 11.54 |
| **29** | 0.0000 | 1.0000 | 0.0000 | 0.00 | 42.61 | 0.00 | 44.11 | 13.29 |
| **30** | 0.0000 | 0.0000 | 1.0000 | 0.00 | 0.00 | 37.75 | 59.49 | 2.77 |

Table S4 BET surface area of selected samples containing single wurtzite phase

| Entry | Cation molar ratio before nitridation | | | BET surface area (m^2^/g) |
| --- | --- | --- | --- | --- |
|  | Zn | Ga | Ge |  |
| **3** | 0.5556 | 0.4444 | 0.0000 | 4.0899 |
| **7** | 0.4750 | 0.2750 | 0.2500 | 11.9475 |
| **8** | 0.4750 | 0.2750 | 0.2500 | 5.8708 |
| **11** | 0.4375 | 0.3125 | 0.2500 | 12.9274 |
| **12** | 0.4375 | 0.3125 | 0.2500 | 5.7576 |
| **16** | 0.4000 | 0.3500 | 0.2500 | 5.4980 |
| **17** | 0.3750 | 0.4375 | 0.1875 | 3.3873 |
| **20** | 0.3300 | 0.4700 | 0.2000 | 6.1780 |
